# Complete genome sequence of the *Microbacterium rhizosphaerae* type strain KACC 19337^T^

**DOI:** 10.1128/mra.00345-24

**Published:** 2024-05-24

**Authors:** Hyorim Choi, Yiseul Kim, Yonghoon Lee, Jun Heo

**Affiliations:** 1 Agricultural Microbiology Division, National Institute of Agricultural Sciences, Rural Development Administration, Wanju-gun, Jeollabuk-do, South Korea; 2 Division of Biotechnology, Jeonbuk National University, Iksan-si, Jeollabuk-do, South Korea; University of Southern California, Los Angeles, California, USA

**Keywords:** genome, *Microbacterium*, type strain, sequence

## Abstract

We report the whole genome sequence of *Microbacterium rhizosphaerae* KACC 19337^T^. The genome consists of a 4.05-Mb circular chromosome, with a G + C content of 69.7 %, and 3,627 total coding genes predicted.

## ANNOUNCEMENT

The genus *Microbacterium*, isolated from various ecological habitats such as the rhizosphere, plants, air, and larvae, was first reported in 1919 (1). Among them, *M. rhizosphaerae* was isolated in 2016 from the rhizosphere soil of Ginseng cultivation in South Korea and proposed as a novel species, described as a Gram-positive, short rod-shaped, and non-motile bacterium with pigmented colonies on Nutrient Agar (NA) medium (2). To date, there has been no genomic information on this species, which was first reported in 2016. For the genome database construction project deposited in the Korean Agricultural Culture Collection (KACC), we performed genome analysis.

Here, we report the whole genome sequence of *Microbacterium rhizosphaerae* KACC 19337^T^. It was deposited into KACC in 2017 and we obtained it from the KACC. It was cultured on NA medium (BD Difco, New Jersey, USA) with pH 6.0 at 28°C for 2 days under aerobic conditions. According to the manufacturer’s protocol, the Qiagen MagAttract HMW DNA Kit (Qiagen, Hilden, Germany) was used for genomic DNA extraction. The DNA products generated from the previous procedure were used for genome sequence analysis on an Illumina NovaSeq 6000 (Illumina, California, USA) and PacBio Sequel IIe (Pacific Biosciences, California, USA). Genomic DNA libraries were created using the TruSeq Nano DNA High Throughput Library Prep Kit (Illumina, California, USA). A total of 10 µL libraries were prepared as 7–12 kb size templates for the PacBio SMRTbell Prep Kit 3.0. Then, they were analyzed using the Sequel II Bind Kit 3.2 and Int Ctrl 3.2. Sequencing was carried out using Sequel II Sequencing Kit 2.0 and SMRT cell 8M trays. HIFI reads were obtained from the PacBio Sequel IIe system and assembled using the microbial assembly application in SMRTlink 11.0.0.146107, based on the Hierarchical Genome Assembly Process (3). The Illumina raw reads of which 90% of the bases had a phred score of 30 or higher were filtered, and adapter trimming was performed using Trimmomatic 0.38 (4). Then the assembly, initially constructed with long-read data, was revised through Pilon v1.21 to correct errors and enhance precision (5). Gene prediction and annotation were conducted by the NCBI Prokaryotic Genome Annotation Pipeline v6.4 (6). All tools were executed with default parameters unless stated otherwise.

The Illumina Novaseq 6000 and PacBio Sequel IIe sequencing generated 34,043,632 read pairs (2 × 150 bp) and 63,660 long reads (609,266,831 bp; 11,307 *N*_50_), respectively. The genome of *M. rhizosphaerae* KACC 19337^T^ consists of a single circular chromosome (4,057,055 bp with G + C content of 69.7%) and no plasmid. The contig resulted in 100% coverage and a depth of 775.95×. The contig assembly contains 3,627 total coding sequences, 6 rRNA genes, 46 tRNA genes, 3 non-coding RNA genes, and 18 pseudogenes.

## Data Availability

The complete genome of *Microbacterium rhizosphaerae* KACC 19337^T^ was deposited in NCBI GenBank under the BioProject accession number PRJNA1044464, the BioSample accession number SAMN38393746, and the GenBank accession number CP139368. The raw Illumina and PacBio reads can access the NCBI's SRA accession numbers SRR26937449 and SRR26937450, respectively.

## References

[1] Orla-Jensen S. 1919. The lactic acid bacteria. Høst and Son, Copenhagen.

[2] Cho SJ, Lee SS. 2017. Microbacterium rhizosphaerae sp. nov., isolated from a Ginseng field, South Korea. Antonie Van Leeuwenhoek 110:11–18. doi:10.1007/s10482-016-0768-4 27688210

[3] Chin CS, Alexander DH, Marks P, Klammer AA, Drake J, Heiner C, Clum A, Copeland A, Huddleston J, Eichler EE, Turner SW, Korlach J. 2013. Nonhybrid, finished microbial genome assemblies from long-read SMRT sequencing data. Nat Methods 10:563–569. doi:10.1038/nmeth.2474 23644548

[4] Bolger AM, Lohse M, Usadel B. 2014. Trimmomatic: a flexible trimmer for illumina sequence data. Bioinformatics 30:2114–2120. doi:10.1093/bioinformatics/btu170 24695404 PMC4103590

[5] Walker BJ, Abeel T, Shea T, Priest M, Abouelliel A, Sakthikumar S, Cuomo CA, Zeng Q, Wortman J, Young SK, Earl AM. 2014. Pilon: an integrated tool for comprehensive microbial variant detection and genome assembly improvement. PLoS One 9:e112963. doi:10.1371/journal.pone.0112963 25409509 PMC4237348

[6] Tatusova T, DiCuccio M, Badretdin A, Chetvernin V, Nawrocki EP, Zaslavsky L, Lomsadze A, Pruitt KD, Borodovsky M, Ostell J. 2016. NCBI prokaryotic genome annotation pipeline. Nucleic Acids Res 44:6614–6624. doi:10.1093/nar/gkw569 27342282 PMC5001611

